# The B Chromosomes of *Prochilodus lineatus* (Teleostei, Characiformes) Are Highly Enriched in Satellite DNAs

**DOI:** 10.3390/cells10061527

**Published:** 2021-06-17

**Authors:** José Henrique Forte Stornioli, Caio Augusto Gomes Goes, Rodrigo Milan Calegari, Rodrigo Zeni dos Santos, Leonardo Moura Giglio, Fausto Foresti, Claudio Oliveira, Manolo Penitente, Fábio Porto-Foresti, Ricardo Utsunomia

**Affiliations:** 1 Faculty of Sciences, São Paulo State University, Bauru, São Paulo 17033-360, Brazil; jose.henrique@unesp.br (J.H.F.S.); caio.goes@unesp.br (C.A.G.G.); milan.calegari@unesp.br (R.M.C.); rodrigo.zeni@unesp.br (R.Z.d.S.); leonardo.moura@unesp.br (L.M.G.); fp.foresti@unesp.br (F.P.-F.); 2 Department of Structural and Functional Biology, Institute of Biosciences, São Paulo State University, Botucatu, São Paulo 18618-689, Brazil; f.foresti@unesp.br (F.F.); claudio.oliveira@unesp.br (C.O.); 3 Mato Grosso State University, Cuiabá, Mato Grosso 78400-000, Brazil; manolopenitente@gmail.com; 4 Department of Genetics, Institute of Biological Sciences and Health, Rural Federal University of Rio de Janeiro, Seropedica, Rio de Janeiro 23897-000, Brazil

**Keywords:** *Prochilodus*, Curimbatá, Cytogenomics, repetitive DNA, supernumerary chromosomes, concerted evolution, Teleostei

## Abstract

B or supernumerary chromosomes are dispensable elements that are widely present in numerous eukaryotes. Due to their non-recombining nature, there is an evident tendency for repetitive DNA accumulation in these elements. Thus, satellite DNA plays an important role in the evolution and diversification of B chromosomes and can provide clues regarding their origin. The characiform *Prochilodus lineatus* was one of the first discovered fish species bearing B chromosomes, with all populations analyzed so far showing one to nine micro-B chromosomes and exhibiting at least three morphological variants (Ba, Bsm, and Bm). To date, a single satellite DNA is known to be located on the B chromosomes of this species, but no information regarding the differentiation of the proposed B-types is available. Here, we characterized the satellitome of *P. lineatus* and mapped 35 satellite DNAs against the chromosomes of *P. lineatus*, of which six were equally located on all B-types and this indicates a similar genomic content. In addition, we describe, for the first time, an entire population without B chromosomes.

## 1. Introduction

Repetitive portions of eukaryotic genomes can be classified into dispersed elements, such as transposable elements and tandemly arrayed sequences, which encompass multigene families and satellite DNAs (satDNAs) [1,2]. With the exception of the multigene families, repetitive DNAs constitute a heterogeneous collection of sequences that differ in organization, composition, and location but have no clear functions [1,3].

SatDNAs are monomeric units with variable sizes arranged in a head-to-tail fashion that can span up to millions of repetitions primarily located in, but not restricted to, centromeric and pericentromeric heterochromatin [4,5,6]. Every species carries a catalog of satDNAs that exhibit a wide diversity of sequence families and usually emerge independently from distinct genomic regions by de novo duplications, occasionally followed by clustering [7]. As the main components of heterochromatin, satDNAs are prone to accumulation in regions of low recombination (or recombination-free), which include sex and B chromosomes due to processes that are analogous to Muller’s ratchet [4,5,8,9,10].

B chromosomes are selfish supernumerary elements that usually do not obey the Mendelian laws of inheritance [9]. They typically arise from the A chromosomes of their species by chromosomal breakage and accumulate in a natural population by drive mechanisms (e.g., when transmission rates of chromosomes are higher than 0.5) [9,11]. Repetitive DNA plays a key role in the differentiation of B chromosomes (due to their non-recombining nature) and can also provide clues regarding the origin of these elements [12,13,14,15,16,17,18].

*Prochilodus lineatus* is a migratory characiform fish with a diploid number of 54 chromosomes. In addition, all populations of *P. lineatus* analyzed to date exhibit a B chromosome system composed of at least three micro-B variants [14,19,20,21]. Likewise, congeneric species also show similar micro-B chromosomes and it is hypothesized that these elements originated in the last common ancestor of several *Prochilodus* species [22,23]. In a pioneering study, Jesus et al. [24] isolated two pericentromeric satDNAs from the genome of *P. lineatus* (SATH1 and SATH2) out of which one (SATH1) was also clustered on the B chromosomes, suggesting an intraspecific origin for these elements [14,24]. This is one of the most well-studied B chromosome systems in fish [14,19,20,21,25,26,27,28,29] and the knowledge on its molecular composition is restricted to a single satDNA.

In recent years, the massive characterization of DNA repeats has become feasible owing to the development of next generation sequencing technologies and associated pipelines [7,30,31,32]. Here, we sequenced the genome of a *P. lineatus* specimen carrying B chromosomes and characterized its satellitome by combining computational and cytogenetic tools, with a focus on the accumulation of satDNAs on the B chromosomes of this species.

## 2. Materials and Methods

### 2.1. Ethics

The procedures for sampling, maintenance, and analysis of the animals were carried out in accordance with the international rules on animal experimentation followed by the Ethics Committee on the Use of Animals at the São Paulo State University (IBB/UNESP), protocol 1204-CEUA/2019. Animals were collected in accordance with Brazilian environmental protection legislation (Collection Permission MMA/IBAMA/SISBIO—number 3245).

### 2.2. Sampling, Chromosomal Preparations, and Genomic DNA Isolation

Live specimens of *P. lineatus* were collected in two distinct sampling points. Looking for individuals bearing the three described B-types, we collected nine individuals (F1 from wild-caught *P. lineatus* collected in the Sapucaí river at the Upper Paraná River basin, 20°34′20.669″ S 47°46′57.868″ W) in the tanks of a fish farm in Sales de Oliveira, São Paulo, Brazil. We also collected 20 specimens of *P. lineatus* in the Apa river (Paraguay River basin, 22°14′58.96″ S 56°55′36.728″ W). Fin clips from all individuals were fixed in 95% ethanol. Mitotic chromosomes were obtained from kidney cells following the protocol established by Foresti et al. [33] (Sapucaí river) and by Foresti et al. [34] (Apa river). After analysis, specimens were fixed in 10% formaldehyde, preserved in 70% ethanol, and deposited in the fish collection of the Fish Genetics Laboratory, Faculty of Sciences, São Paulo State University in Bauru-SP under the voucher numbers LG13393-LG13407 (Sapucaí river) and LG13501-13520 (Apa river).

In order to characterize the satellitome of *P. lineatus*, we selected one specimen carrying three B chromosomes and extracted genomic DNA from fin clips using the Wizard Genomic DNA Purification Kit (Promega) following the manufacturer’s instructions. After that, we checked DNA quality with NanoDrop (Thermo Fisher Scientific) and 1% agarose gel electrophoresis.

### 2.3. Genome Sequencing and Bioinformatic Analyses

We sequenced gDNA from a *P. lineatus* specimen on the BGISEQ-500 platform (paired-end 2 × 100 bp) at BGI (BGI Shenzhen Corporation, Shenzhen, China). Raw reads are available in the sequence read archive (SRA-NCBI) under the accession number SRR11676686. In total, we sequenced 3.64 Gb, resulting in a sequencing coverage of approximately 2.14×, under the assumption that the genome of *P. lineatus* has an estimated size of 1.7 Gb [35].

After quality and adapter trimming with Trimmomatic [35], we characterized the satDNAs of *P. lineatus* using several iterations of the TAREAN tool [36] followed by filtering the identified satDNAs with DeconSeq [37] until no further satDNA was found. Thereafter, we performed additional iterations with the RepeatExplorer1 software [30], followed by filtering the discovered satDNAs with DeconSeq until no satDNA was found (satMiner protocol). Next, we filtered and removed other tandemly repeated elements, such as multigene families, and performed a homology search to group the sequences into the same sequence variant, family, and superfamily if their identities were greater than 95%, 80%, or 50%, respectively. We then estimated divergence and abundance values using the RepeatMasker software upon a random selection of 2 × 5,000,000 reads [38]. In this analysis, we mapped the selected reads against the whole catalog of satDNAs so that the number of mapped reads divided by the total number of analyzed nucleotides would indicate the relative abundances of each satDNA. At the same time, the divergence values are represented as weighted average Kimura divergence values for each repeat family. The satDNA families were named according to Ruiz-Ruano et al. [7], with each satDNA beginning with a species abbreviation (e.g., Pli for *P. lineatus*) followed by the term “Sat” and a catalog number in order of decreasing abundance followed by the consensus monomer length. Each satDNA was deposited in GenBank with accession numbers MZ161094-MZ161143. Furthermore, consensus sequences for each of the 51 satDNA families were manually analyzed to design PCR primers. If the monomer of a given satDNA was shorter than 30 nt, we did not design primers and this resulted in a total of 38 primer pairs to be amplified in PCR reactions.

Since four characiform species already had their satellitomes characterized, we BLAST-searched [39] the satellitome of *P. lineatus* against the NCBI nucleotide collection to investigate whether there were any conserved satDNA families within this order. To perform this, we filtered the results by excluding alignments smaller than the size of the satellite monomers and aligned the consensus sequences using MUSCLE [40].

The above-mentioned analysis identified several satDNAs (*n* = 8; PliSat12, PliSat15, PliSat18, PliSat21, PliSat30, PliSat36, PliSat40, and PliSat43) in *Megaleporinus macrocephalus* (described below) and one (PliSat26) in all the Characiformes species, previously known as CharSat01 [41]. We then downloaded genomic libraries from *M. macrocephalus* (SRA accession number: SRR7263034) to perform comparative analyses. First, we generated multiple satDNA coverage depths and sequence variant profiles using the RepeatProfiler pipeline [42] to visualize their tandem organization and sequence variation in the referred genomes. In order to perform this, we first randomly selected 2 × 1,000,000 reads (2 × 101 nt) from both species. We then concatenated monomers of the target satDNAs to a minimum of 200 nt and text-provided ten single-copy fish genes to be mapped for single-copy normalization of the read coverage (*ppfia1* (XM_022685633.1), *foxl2* (XM_007232295.3), *prospero* (XM_017708821.1), *msh4* (XM_017711771.1), *zdhhc22* (XM_017711775.1), *coq6* (XM_017711829.1), *znf106* (XM_017711848.1), *lactamase* (XM_022682177.1), *gastrula zinc finger* (XM_022685636.1), and *tubulin-kinase* (XM_017711762.1)), as described by dos Santos et al. [41]. Read mapping was performed with Bowtie2 [43] with the preset values “-sensitive” and “-no-mixed.” 

The genomic abundances and Kimura 2-parameter divergence values were estimated independently by sampling 2 × 1,000,000 reads (2 × 101 nt) from both species and aligning them to the eight shared satDNAs. Reads of both species were separately mapped against concatenated monomers of satDNA consensus sequences (spanning 200 nucleotides) using RepeatMasker. Next, we used the calcDivergenceFromAlign.pl script from the RepeatMasker utils and created repeat landscapes so that the number of mapped reads divided by the total of analyzed nucleotides would indicate the relative abundances of each satDNA (represented on the y-axis of the landscape). In contrast, the divergence between the mapped reads and consensus sequences would indicate the Kimura-2-parameter distances (further represented in bins of zero to 70%). Thus, copies with low divergence bins are very similar to the consensus sequence. 

### 2.4. Fluorescent In Situ Hybridization (FISH)

Prior to FISH experiments, 35 satDNA probes were labeled with digoxigenin-11-dUTP in PCR reactions. FISH was performed using the method described in Pinkel et al. [44] with small modifications as described in Utsunomia et al. [45]. Briefly, chromosomes were treated with RNAse (50 μg/mL) for 50 min, followed by chromosomal DNA denaturation in 70% formamide/2× SSC for 2 min at 70 °C. Post-hybridization, slides were washed in 0.2× SSC/15% formamide for 20 min at 42 °C, followed by a second wash in 0.1× SSC for 15 min at 60 °C, and a final wash in 4× SSC/0.5% Tween for 10 min at room temperature. Probe detection was performed with anti-digoxigenin-rhodamine (Roche) and the chromosomes were counterstained with DAPI (4′, 6-diamino-2-phenylindole, Vector Laboratories) and analyzed using an optical microscope (Olympus BX61). Images were captured with the DP Controller software (Olympus). A minimum of 10 cells from each individual were analyzed to confirm the observed FISH patterns.

In order to locate multiple probes in the same metaphase plate, especially those mapping simultaneously in A and B chromosomes (e.g., PliSat03, PliSat04, and PliSat05), we performed multiple rounds of FISH with the mentioned probes. In this case, we first performed a regular FISH with a single probe and captured several images. Next, we removed the coverslips and washed the slides with 4× SSC/1% Triton-100 for 5 min. Thereafter, we proceeded with the hybridization protocol again with a reduction of 70% formamide/2× SSC and denaturation time to 30 s. Using this approach, we could successfully map the satDNA sequences for five consecutive rounds.

## 3. Results

### 3.1. Karyotypes and the First Description of a Population without B Chromosomes

**Figure 1:**
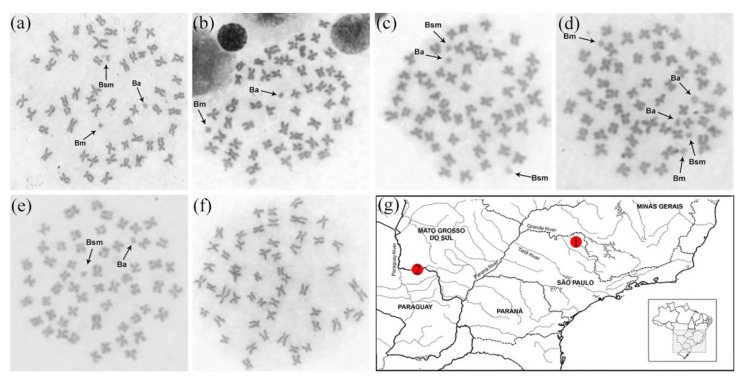
Metaphase plates and collection points. (**a**–**e**): metaphase plates from individuals collected in the Sapucaí river and evidences the occurrence of distinct B-types; (**f**) example of metaphase plate from individuals collected in the Apa river and evidences the absence of B chromosomes; (**g**) map indicating the sampling points: (1) Sapucaí river; (2) Apa river.

All the *P. lineatus* specimens analyzed exhibited a diploid chromosome number of 2*n* = 54 and were primarily metacentric and submetacentric chromosomes. In addition, mitotically stable micro-B chromosomes were identified in all individuals collected from the Sapucaí river, as expected (Penitente unpublished data). The B chromosome numbers per cell varied between one and five morphologically distinguishable types, including three variants which are metacentric (Bm), submetacentric (Bsm), and acrocentric (Ba), as described previously (Figure 1) [21]. Remarkably, B chromosomes were not identified in the Apa River population (Figure 1), which is the first report of a *P*. *lineatus* population without B chromosomes.

### 3.2. The B chromosomes of P. lineatus Are Highly-Enriched in Satellite DNAs

After three iterations of TAREAN and one iteration using the RepeatExplorer1 protocol, we found 51 satDNA families for *P*. *lineatus*, with the repeat unit lengths ranging from 6 to 3008 bp, with a median of 59 bp (Table 1). The A + T content of the satDNA families varied from 40% to 76.2%, with a mean value of 55.24%, and 39 of the satellites showed an A + T content greater than 50%. The homology search for the *P*. *lineatus* satDNA families revealed two superfamilies (SF1—PliSat01 and PliSat09; SF2—PliSat33 and PliSat42). Among the 51 satDNA families, we designed 38 primer pairs (Table S1) out of which 35 yielded ladder-like patterns after PCR reactions (Table S1) and confirmed their tandem organization. In total, 35 FISH probes were constructed; after the FISH experiments, only eight showed a clustered organization in the *P*. *lineatus* chromosomes, whereas the remaining satDNAs were considered non-clustered at the chromosomal level.

**Figure 2:**
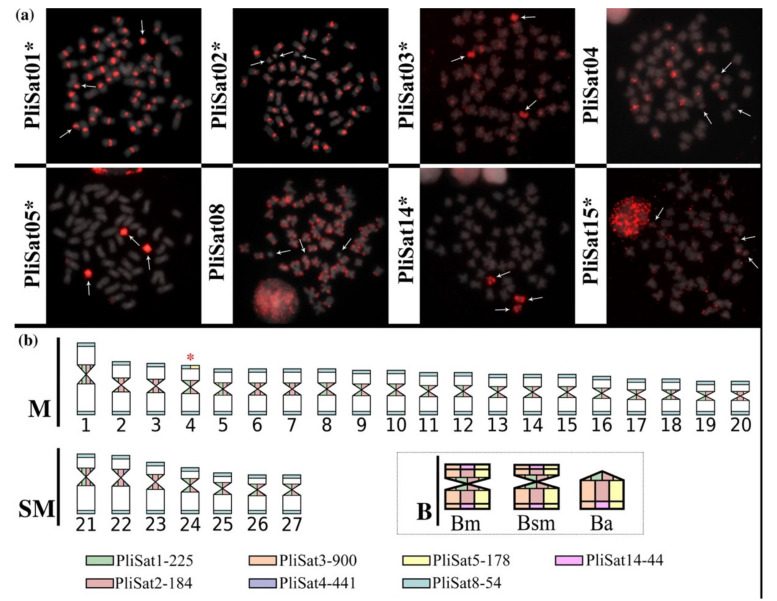
Chromosomal location of satellite DNA sequences. (**a**) Metaphase plates containing the three described B-types after FISH with different satDNA probes and evidences the similar hybridization patterns for all tested probes. (**b**) Idiogram of *P*. *lineatus* karyotype highlighting the distribution of different satDNA families. The B-types shares all the satDNAs (gray box) and are probably derived from pair No. 4 (red asterisk).

Primarily, clustered satDNAs were observed in the centromeric or telomeric regions in addition to those observed exclusively in the B chromosomes (Figure 2). Importantly, we did not notice any difference in the clustering patterns of satDNAs on the distinct *P*. *lineatus* B-variants. Finally, the satDNA distribution in the Apa river individuals was found to be similar to that observed in individuals from the Sapucaí river, with the exception of PliSat14 which was exclusively mapped on the B chromosomes (Figure 2 and Figure S1).

### 3.3. P. lineatus and M. macrocephalus Share Several satDNAs

**Figure 3:**
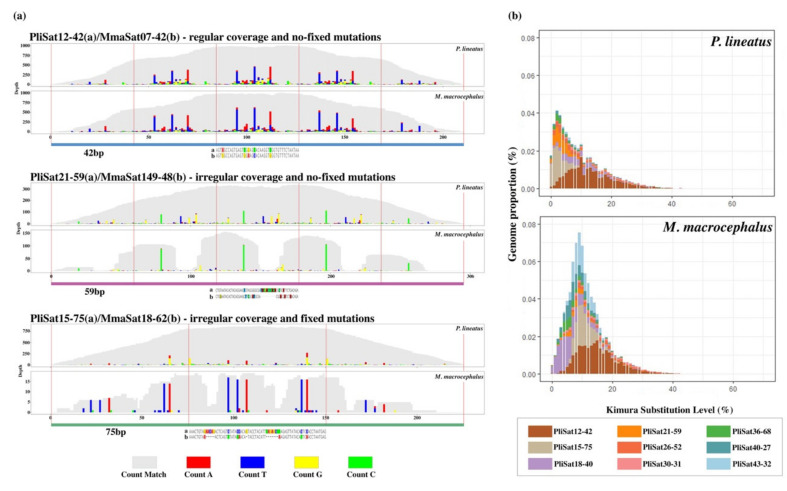
Tandem repeat organization of multiple satellite DNAs in distantly-related species. (**a**) Examples of variant profiles found for three distinct satDNA families against a consensus sequence. The alignments between consensus satDNA sequences are below the variant profiles (**b**) Repeat landscapes of the nine shared satellite DNAs detected in the sequenced reads of *P*. *lineatus* and *M*. *macrocephalus*. Color-coded bar plots are based on RepeatMasker showing K2P distance to their corresponding consensus sequence on the x axis and the abundance on the y axis.

BLAST searches against the NCBI database nucleotide collection revealed multiple significant hits. Among them, we found the following: (i) One 52 nt-long satDNA (PliSat26) was shared among *M. macrocephalus*, *Characidium gomesi*, and *Astyanax paranae* and this sequence was previously known as CharSat01-52 and is conserved within the Characiformes clade [41]; (ii) one satDNA (PliSat8) was shared with *Semaprochilodus taeniurus* (Figure S2) and this sequence was previously characterized in the W chromosome of the referred species (known as STW4, accession number: JX157128.1; [46]) and seemed conserved in the family Prochilodontidae; (iii) eight satDNAs (PliSat12, PlisSat15, PliSat18, PliSat21, PliSat30, PliSat36, PliSat40, and PliSat43) were shared with *M*. *macrocephalus* (Figure 3, Figure S2), indicating that these tandem repeats originated before the split of Prochilodontidae and Anostomidae, which also includes two other characiform families (Chilodontidae and Curimatidae) with an approximate divergence time of 50–70 million years according to Kolmann et al. [47].

The alignments of eight consensus satDNAs of *P*. *lineatus* and *M*. *macrocephalus* revealed high sequence conservation and the RepeatProfiler and RepeatMasker analyses added new information in this case. Primarily, we observed no consensus sequences of satDNA families showing 100% identity in the alignments between species (Figure 3). However, the variant profiles provided a genome-wide view of the organization of satDNAs and revealed that six satDNAs (PliSat12, PliSat18, PliSat21, PliSat30, PliSat36, and PliSat40) did not present a fixed mutation (indel or substitution) for a specific species. In this context, even the large gaps observed in the consensus alignments (e.g., PliSat21-59/MmaSat16-51) are not fully fixed (Figure 3a) after millions of years of divergence [47]. Interestingly, the variant profiles of PliSat12-42/MmaSat07-42 revealed that this satDNA was highly abundant in both genomes and also exhibited a notable similarity in the SNPs. Finally, the variant profiles of PliSat15-75/MmaSat18-62 and PliSat43-32/MmaSat26-22 showed a higher degree of differentiation with fixed mutations (Figure 3a).

Repeat landscapes were constructed for the above-mentioned conserved satDNAs; these showed that the abundance of sequence variants fluctuates between species, which is evidently noted for PliSat18-40/MmaSat16-51 and PliSat15-75/MmaSat18-62 (Figure 3b). However, we can also note the similar shape of PliSat12-42/MmaSat07-42 in the repeat landscape according to our previous analysis of variant profiles.

## 4. Discussion

### 4.1. The Karyotypes of P. lineatus Populations

The chromosomes of the *Prochilodus* species have been extensively analyzed in the last 40 years and a conserved karyotype structure with 2*n* = 54 chromosomes has been reported to date and is primarily composed of metacentric and submetacentric chromosomes [23,28,48,49]. Remarkably, several *Prochilodus* species exhibit a micro-B chromosome system, which is assumed to have emerged once in this group diverged into multiple B-variants in distinct lineages over time [21,22,23,28,50]. Our results corroborate the common origin of the three observed B-types in *P. lineatus*, despite it showing morphological differences, all carry the same satDNAs with similar hybridization patterns.

The accumulation of DNA repeats on B chromosomes is a common feature because of their usual non-recombining nature and the predominance of heterochromatic regions [9,11]. Considering that the three B-types showed very similar hybridization patterns for all satDNAs and that PliSat05 was highly accumulated on these elements and in a single pair of chromosomes (pair No. 4; Figure S3), we suggest that the B chromosome origin of *P. lineatus* is related to this chromosome pair. Therefore, a detailed genome-wide analysis of gene content and diversity will permit a better understanding of the B-types, especially because they show different transmission rates [21].

Here, we also describe for the first time an entire population of *P. lineatus* collected from the Paraguay River basin, which does not exhibit B chromosomes. The exclusive and restricted occurrence of B chromosomes in certain populations of a single species is quite common and has been reported multiple times in many fish species [17,51,52]. In general, this is often attributed to the low vagility of these species, in which populations are geographically isolated and evolve independently [17]. Thus, the case presented herein is noteworthy, since *P. lineatus* is considered one of the most important migratory fish from the Neotropical region [53] and previous analyses indicated that this species has been maintained as a single variable stock in the Paraná system, which includes both of our collection points [54,55,56]. A hypothesis that could explain the absence of genetic diversity in this species in such a large river basin, such as that of the Paraná River, could be related to the molecular markers (e.g., mitochondrial sequences and microsatellites) in most population genetics studies of this species. Melo et al. [57] revealed little divergence among the mitochondrial lineages in *Prochilodus*, suggesting that recent episodes of diversification would explain the observed patterns.

The intrapopulation and interpopulation variation of the B-frequency is a common feature and previous studies have shown a correlation between the presence of B chromosomes and environmental conditions, such as temperature, altitude, and rainfall [58,59,60] as well as seasonal cycles [61]. It is well-known that B chromosomes of multiple species carry active single-copy genes as well as influence A-located gene expression, while satDNAs have been widely described as important gene regulators [41,62,63,64,65,66,67,68]. Thus, one hypothesis is that the correlation of B presence with the environment could be due to selective constraints (in favor of or against the presence of B chromosomes).

### 4.2. The Satellitome of Prochilodus lineatus

Here, we described 51 satDNA families from *P. lineatus*, which is the fifth fish species to have its own characterized satellitome. Remarkably, only eight out of 35 satDNAs were visualized via FISH in *P. lineatus*, indicating that several satDNAs are organized as short arrays in this genome (<10 kb, the boundary of FISH sensitivity). Moreover, two satDNA families (PliSat01 and PliSat02) were co-located on all centromeres. However, they did not show any sequence similarity, indicating that two independently originated satDNAs became the centromeric satDNAs in this species including the A and B chromosomes, which might indicate that these satDNAs play a centromeric function.

Regarding the conservation and persistence of satDNAs between distant species, the sharing of nine satDNAs with *M. macrocephalus* is notable and points to the long-term persistence and conservation of these sequences over millions of years [47]. To date, the long-term evolutionary persistence of satellite DNAs is uncommon and there have been very few described cases [41,67,69,70,71,72] primarily because this type of sequence follows a concerted evolutionary pattern that results in high levels of intraspecies sequence homogeneity and low rates of evolutionary persistence [5,73,74]. Therefore, we suggest that tandem repeats can naturally persist for millions of years without major changes in their profiles or that this specific maintenance could be related to selective constraints. Importantly, we cannot state that the other 42 satDNAs of *P. lineatus* were not present in the genomes of other Characiformes species since we only analyzed the consensus sequences of all species outputted from RepeatExplorer and TAREAN pipelines.

## 5. Conclusions

In this study, we characterized the catalog of satDNAs of *P. lineatus*, which permitted us to confirm the single origin of the B-variants in this species and suggest their origin from a chromosome pair (No. 4) with subsequent morphological diversification. In addition, we found several satDNA families shared between *M. macrocephalus*, indicating unusual long-term conservation of these sequences. Finally, we described a population of *P. lineatus* that does not exhibit B chromosomes for the first time, which could indicate a certain degree of population differentiation of this species in the Paraná River basin or a possible of correlation between B-presence and environmental conditions.

## Figures and Tables

**Table 1:** Main features of the 51 satDNA families found in *P*. *lineatus* genome. Clustered satDNAs are highlighted in gray.

SF	satDNA Family	RUL	A+T (%)	Abundance (%)	Divergence (%)
1	PliSat01-225	225	64.5	1.332 × 10^−2^	7.25
	PliSat02-184	184	54.9	9.882 × 10^−3^	10.89
	PliSat03-900	900	56.5	4.829 × 10^−3^	1.84
	PliSat04-441	441	57.9	3.191 × 10^−3^	6.43
	PliSat05-178	178	64.6	3.097 × 10^−3^	8.99
	PliSat06-44	44	47.8	2.021 × 10^−3^	13.85
	PliSat07-3008	3008	63.4	1.920 × 10^−3^	3.31
	PliSat08-54	54	62.9	1.847 × 10^−3^	4.04
1	PliSat09-495	495	58.0	1.809 × 10^−3^	5.78
	PliSat10-25	25	40.0	1.215 × 10^−3^	11.61
	PliSat11-709	709	50.4	1.120 × 10^−3^	9.90
	PliSat12-42	42	52.4	9.841 × 10^−4^	13.58
	PliSat13-1928	1928	62.4	8.420 × 10^−4^	7.86
	PliSat14-44	44	50.0	5.822 × 10^−4^	7.52
	PliSat15-75	75	61.3	4.322 × 10^−4^	3.33
	PliSat16-16	67	58.2	3.913 × 10^−4^	3.79
	PliSat17-21	21	76.2	3.087 × 10^−4^	15.05
	PliSat18-40	40	57.5	2.978 × 10^−4^	10.47
	PliSat19-30	30	56.7	2.642 × 10^−4^	12.96
	PliSat20-54	54	57.4	2.558 × 10^−4^	9.63
	PliSat21-59	59	49.2	2.382 × 10^−4^	4.69
	PliSat22-37	37	45.9	2.120 × 10^−4^	4.59
	PliSat23-39	39	53.8	1.860 × 10^−4^	10.87
	PliSat24-31	31	51.6	1.846 × 10^−4^	17.13
	PliSat25-34	34	47.1	1.611 × 10^−4^	5.42
	PliSat26-52	52	59.6	1.569 × 10^−4^	8.83
	PliSat27-1683	1683	52.0	1.368 × 10^−4^	2.25
	PliSat28-32	32	56.8	1.361 × 10^−4^	14.45
	PliSat29-60	60	45.0	1.251 × 10^−4^	3.31
	PliSat30-31	31	54.8	1.225 × 10^−4^	16.69
	PliSat31-707	707	53.6	1.188 × 10^−4^	4.27
	PliSat32-30	30	66.3	1.185 × 10^−4^	12.19
2	PliSat33-6	6	57.1	1.040 × 10^−4^	25.51
	PliSat34-39	39	53.8	1.013 × 10^−4^	7.97
	PliSat35-1128	1128	61.0	9.474 × 10^−5^	10.43
	PliSat36-68	68	63.2	9.039 × 10^−5^	5.55
	PliSat37-554	554	50.0	8.823 × 10^−5^	5.47
	PliSat38-32	32	53.1	8.486 × 10^−5^	6.29
	PliSat39-915	915	54.3	7.846 × 10^−5^	1.35
	PliSat40-27	27	48.8	7.708 × 10^−5^	14.00
2	PliSat41-162	162	45.8	7.486 × 10^−5^	1.75
	PliSat42-24	24	55.6	6.696 × 10^−5^	7.75
	PliSat43-32	32	53.1	6.279 × 10^−5^	9.61
	PliSat44-1134	1134	51.6	6.220 × 10^−5^	3.43
	PliSat45-340	340	50.6	6.194 × 10^−5^	4.83
	PliSat46-29	29	65.5	6.009 × 10^−5^	13.97
	PliSat47-574	574	53.8	5.280 × 10^−5^	6.89
	PliSat48-387	387	48.8	4.802 × 10^−5^	5.00
	PliSat49-67	67	49.3	4.517 × 10^−5^	4.30
	PliSat50-48	48	58.3	4.280 × 10^−5^	9.07
	PliSat51-911	911	55.0	3.897 × 10^−5^	5.00

SF: Superfamily. RUL: repeat unit length.

## Data Availability

All data reported in this manuscript were deposited on public database. Raw reads were deposited on the sequence read archive (SRA-NCBI, SRR11676686); each satellite DNA consensus sequence was deposited in the GenBank database (MZ161094-MZ161143).

## Supplementary Materials

The following are available online at https://www.mdpi.com/article/10.3390/cells10061527/s1, Figure S1: Chromosomal location of satellite DNA sequences in the two analyzed populations, note the similar distribution of satDNA families on the A chromosomes, Figure S2: Tandem repeat organization of multiple satellite DNAs in two distantly related species, examples of variant profiles found for three distinct satDNA families against a consensus sequence, Figure S3: Sequential FISH using multiple probes (PliSat03, PliSat04, and PliSat05), Table S1: Designed primers in the present study.
