# Supplementary material for: The B Chromosomes of Prochilodus lineatus (Teleostei, Characiformes) Are Highly Enriched in Satellite DNAs
Source: Cells. 2021 Jun 17;10(6):1527. doi: 10.3390/cells10061527 (PMC8235050; doi:10.3390/cells10061527)
Supplement: Supplementary file 1 [file cells-10-01527-s001.zip › cells-1244575-supplementary/cells-1244575-send proof supplementary/Cells-1244575-Table S1.pdf]

cells

MDPI

Table S1. Designed primers in the present study. Primers highlighted in red did not amplify.

satDNA family	Primer F	Primer R
PliSat01	TGTTTCTAAGCAATAGAAAGTTA	GTCACAAAACAAGTTTCATTAC
PliSat02	GGAAGGTCCTAGAGTTACCA	GTACGATTGGTTTTTCCCCC
PliSat03 (SATH1)	GCTGCAGCAAAAACCCTACC	AGTGGGAGCTAGGGTTAGGG
PliSat04	GGCTCAGGGAAAACCACAAA	GGTCCAGGAGGAGTTTTAGG
PliSat05	GAAGAACCACCCAAAAACGC	GGGAAAATGCGTGCACATGA
PliSat08	GTAAGTAGTGTGTTGGTGTA	CACATTACACTTACTGAGTT
PliSat09	GATCGTTCCATTTCCCATGC	CGAGAGCTCTGCTATACTCC
PliSat11	ATGCGATCGGTATTTATTCCGGG	CACGTCACGTTTAGAGCGGG
PliSat12	ACTCACTGGCCACTTTATTAG	GAAGCACAAGGTGGGTGTTT
PliSat13	AAATACAATAGGGTCCCTGCA	GGTCTTAAAATACTCTGTGGA
PliSat14	GTGAAAGGTGACGTTGCTGCGT	CTTTCACCTTCGGCATTCATATC
PliSat15	CCTAATGAGAAACTGTAGAACAG	GACTCTAAATGTAGGTATTGTGA
PliSat16	ATACTGGATTAAAATCACTCCACAA	GTGTGTGTAGTTTTTACAGTCTGA
PliSat18	GTGTTGGTGATGGATAAAG	CTTCATCCCTTCATTCCTTT
PliSat19	GTAGCAGTAATGCTAACCA	TGCTACTGAGAGCTGATTG
PliSat20	CAATCAAATCCTCACAGCAATG	CATTAGTGAGGTCAGGTACTG
PliSat21	CGATACGACCCACACGCTCA	CCCCCTACACTTCCTGTAGT
PliSat22	CCTCTCTCCCTCACTCTGT	GTTTGGTGTGAGGTGTGTAC
PliSat23	ATAGAGGAGTGAGGGTCAGTAT	GTGTCTAATCCACAGTCATAC
PliSat25	AGACTGGTGCTCCATCCTC	GAGACAGTCAGTACAGAGG
PliSat27	GCTAGACCATATGGGACCAC	TTAAAGCTGAGCCACACGTT
PliSat28	GATGCGGAACTACTGCCCA	CGCATCAGACCACACAGGA
PliSat29	CCGGTGTGTTACTGTAATGTAG	GTATCAGTAGAGACGGGGGT
PliSat31	TACAGCCCTGGAATGGCAAG	GACTAAACAAGCCTACGCCC
PliSat32	GGATTAATCACAGTCTGAG	TCCAGGTTTAGATACTCAG
PliSat34	GTCTGTAATGTTCTGGTGGT	TGCTTAACTGCACAGACTGA
PliSat35	TCAGACCTACAAGGTAAGCC	CTTGTTATTCTGTAGAGGACAC
PliSat36	GCTAAAGTCTGAGTAGACTGA	CTTTAGCAGAGCTCAGTAAC
PliSat37	CTGCTCCAGACGACATAGGTC	GGACAGGGTTACTGGGACAT
PliSat39	CTTGATGGTTTAGTCATAAGCGG	CAGAACTGCTGTTGTTACCTTG
PliSat41	CAGCCCTCACCTTAACTCCA	GGAGGTTATTTTAGTGTTGGAGC
PliSat44	CCGTACTTCTTGCACAATGC	CGTGATCTTCGGTTTACGAGA
PliSat45	GCAAAGAACAGGAACAAGTGG	AACCATGTCAGGAAGCTCGA
PliSat47	GCAGCCTTACCAAAGTCCTT	TAGTAGTGGTGCCTACTGAG
PliSat48	CTGGCTTGATGTCCAATTATTC	TGACTCCCGTGACCTGATAA
PliSat49	CCTGATACCACACCGAGAGA	GTGGTGTTAGTGGTCTGATC
PliSat50	GAGAGAGGAAACACTACTCAG	GAGAAAGATCTAGAACGATAACTG
PliSat51	AAGCCAAGGACACTGAACGA	CACTGGATCTCCTGTTTACTAC
