# Supplementary figures and images for: The B Chromosomes of Prochilodus lineatus (Teleostei, Characiformes) Are Highly Enriched in Satellite DNAs
Source: Cells. 2021 Jun 17;10(6):1527. doi: 10.3390/cells10061527 (PMC8235050; doi:10.3390/cells10061527)

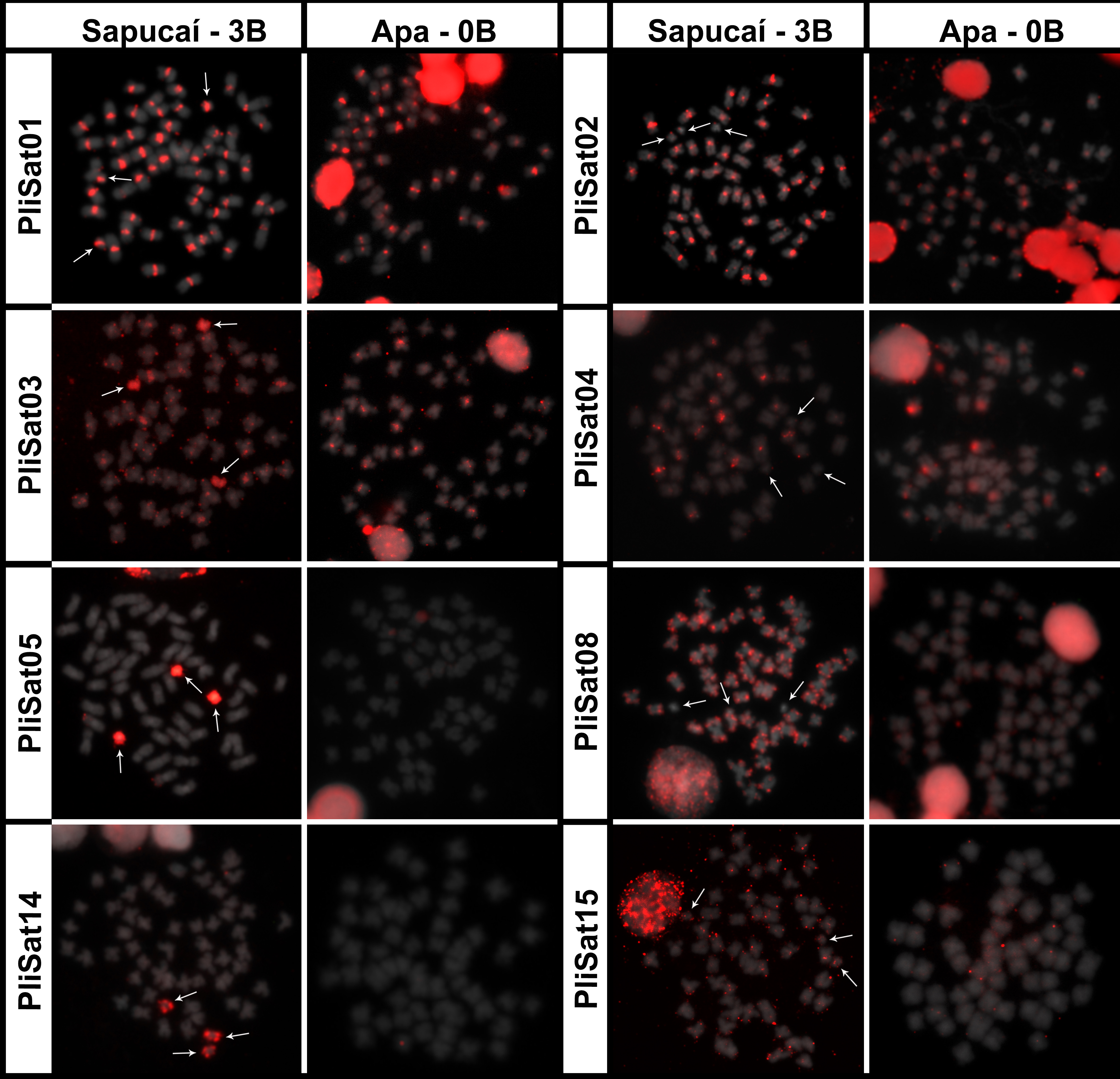

Supplement: Supplementary file 1 [file cells-10-01527-s001.zip › cells-1244575-supplementary/cells-1244575-send proof supplementary/Figure S1.jpg]

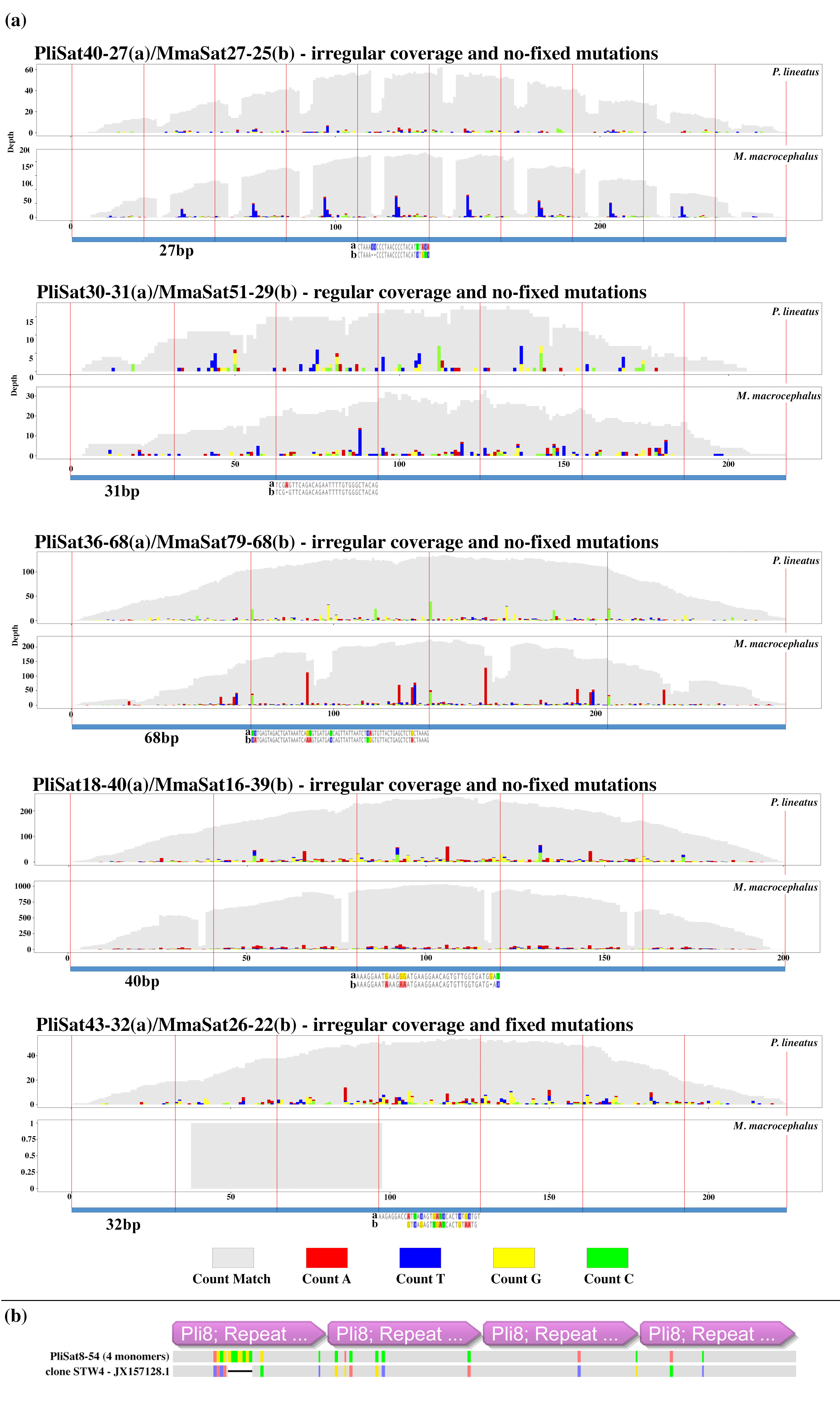

Supplement: Supplementary file 1 [file cells-10-01527-s001.zip › cells-1244575-supplementary/cells-1244575-send proof supplementary/Figure S2.jpg]

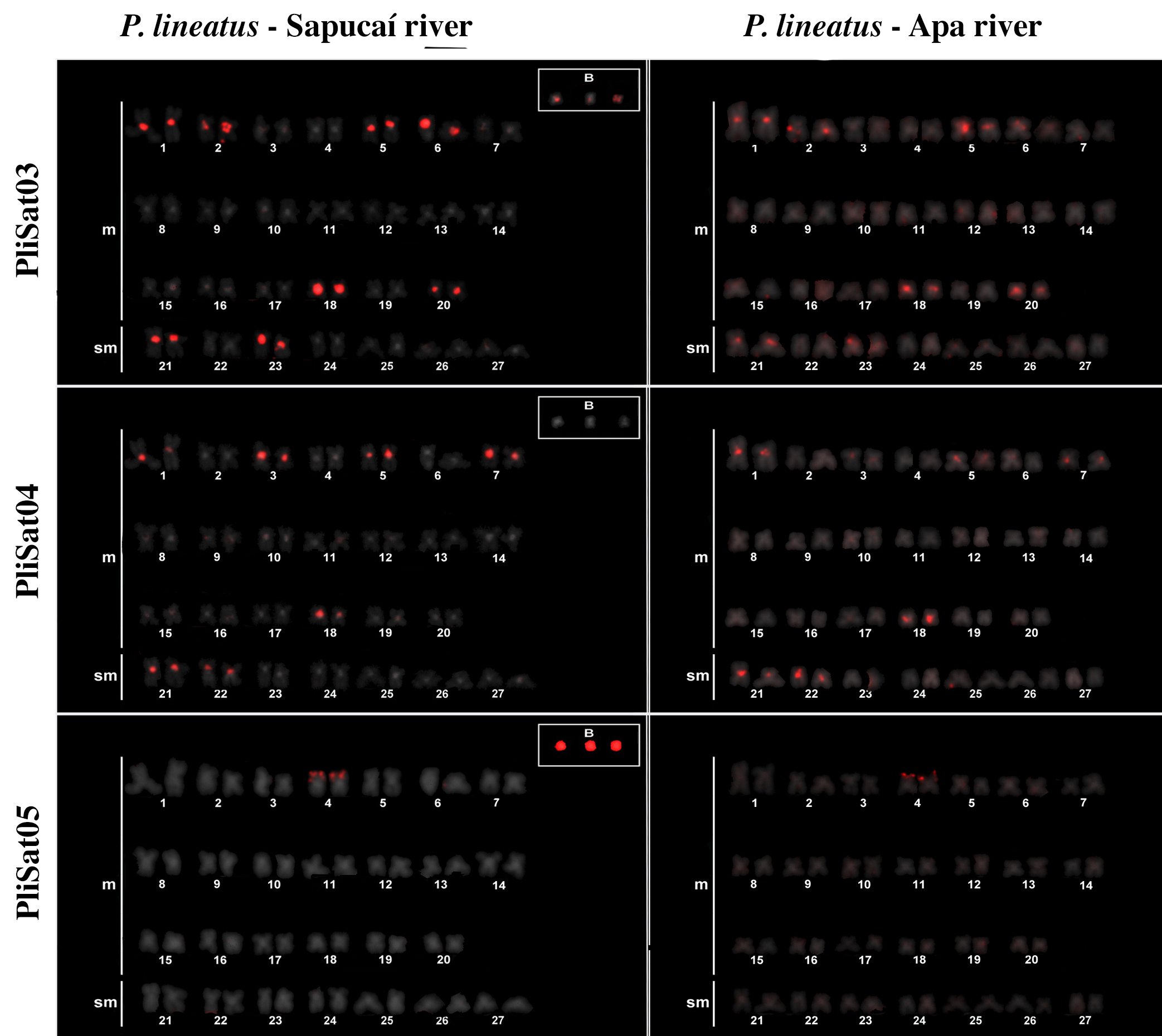

Supplement: Supplementary file 1 [file cells-10-01527-s001.zip › cells-1244575-supplementary/cells-1244575-send proof supplementary/Figure S3.jpg]
